# Development and validation of a questionnaire for assessing visual and auditory spatial localization abilities in dual sensory impairment

**DOI:** 10.1038/s41598-024-58363-6

**Published:** 2024-04-04

**Authors:** Yingzi Xiong, Joseph Paul Nemargut, Chris Bradley, Walter Wittich, Gordon E. Legge

**Affiliations:** 1 Lions Vision Research and Rehabilitation Center, Wilmer Eye Institute, Johns Hopkins University, Baltimore, MD USA; 2 Center for Applied and Translational Sensory Sciences, University of Minnesota, Minneapolis, USA; 3 School of Optometry, Université de Montréal, Montreal, Canada

**Keywords:** Psychology, Health care

## Abstract

Spatial localization is important for social interaction and safe mobility, and relies heavily on vision and hearing. While people with vision or hearing impairment compensate with their intact sense, people with dual sensory impairment (DSI) may require rehabilitation strategies that take both impairments into account. There is currently no tool for assessing the joint effect of vision and hearing impairment on spatial localization in this large and increasing population. To this end, we developed a novel Dual Sensory Spatial Localization Questionnaire (DS-SLQ) that consists of 35 everyday spatial localization tasks. The DS-SLQ asks participants about their difficulty completing different tasks using only vision or hearing, as well as the primary sense they rely on for each task. We administered the DS-SLQ to 104 participants with heterogenous vision and hearing status. Rasch analysis confirmed the psychometric validity of the DS-SLQ and the feasibility of comparing vision and hearing spatial abilities in a unified framework. Vision and hearing impairment were associated with decreased visual and auditory spatial abilities. Differences between vision and hearing abilities predicted overall sensory reliance patterns. In DSI rehabilitation, DS-SLQ may be useful for measuring vision and hearing spatial localization abilities and predicting the better sense for completing different spatial localization tasks.

## Introduction

Dual sensory impairment (DSI) refers to combined vision and hearing impairment. Worldwide, 0.2% of the population have severe DSI (i.e., they are deafblind), and a larger percentage has milder forms with residual functional vision and hearing^1^. In the United States, it is estimated that approximately 40% of people visiting low vision clinics also have hearing impairment^2,3^. Despite being a large and increasing percentage of the population, individuals with DSI have received less attention than people with single sensory impairment in the daily challenges they face, and the special rehabilitation approaches they need^4–6^. While vision and hearing rehabilitation have each achieved success in addressing challenges in their own specialties, the functional difficulties of DSI cannot be adequately addressed by two separate rehabilitation systems^7,8^.

DSI affects independent physical and social functioning. Compared to healthy individuals, or those with single sensory impairment, people with DSI are less likely to participate in physical and social activities and more likely to show depression symptoms^9–11^. A fundamental prerequisite for functional independence is spatial localization—the ability to judge the direction and distance of people and objects around us^12^. In everyday life, spatial localization is important for both safe navigation and effective social interactions. For example, when crossing a street, we need to determine the locations of cars and pedestrians around us; at a social event, we need to locate other attendees to behave effectively. While senses other than vision and hearing, as well as mobility aids, can assist with spatial localization to a certain extent, vision and hearing remain the most important senses for locating objects beyond arm’s reach^12^.

Both vision and hearing impairment affect spatial localization. Individuals with vision impairment frequently report difficulties in spatial localization^13–15^, while impaired binaural hearing makes it challenging to orient to an object in space or determine the direction of a sound^15–19^. Vision and hearing interact with each other ubiquitously in our daily lives and compensate for each other when one or the other sense is impaired. For people with vision impairment, using hearing to compensate is a key principle of their Orientation & Mobility (O&M) training^12^. For people with hearing impairment, using vision to compensate for deficits in sound directionality is also critical, as hearing aids primarily focus on preserving speech quality^7,8,13^. These compensatory strategies may no longer apply to a person with both hearing and vision impairments. For example, in the O&M field clinicians frequently report that their patients rely too little or too much on their residual vision. To develop individualized rehabilitation strategies for an individual with DSI, it is important to understand not only the modality-specific effects of vision and hearing impairment, but also what the better sense is and which sense the individual tends to rely on to perform different tasks.

There is currently no clinical tool available for jointly assessing the vision and hearing spatial localization abilities of individuals with DSI, nor is there a tool that enables direct comparison of such abilities on the same measurement scale. Laboratory tasks often measure visual, auditory, or audiovisual spatial localization performance using simple stimuli in controlled environments to understand the integration of vision and hearing^15,20–22^, but such simplified tasks do not represent real-life situations. In vision and hearing clinical practice, patient functioning in real-life contexts is often assessed by self-reported difficulties in common daily activities. However, existing questionnaires are specific to vision or hearing and do not support the comparison of the two modalities^12,23,24^. The interRAI Community Health Assessment is thus far the only survey tool developed for people with DSI, which uses a single question with a yes/no response to explore whether deafblind clients have difficulties with locating sounds^24^. The Speech Spatial Qualities questionnaire was originally developed for individuals with hearing impairment and consists of items related to sound localization^23^. It was later adapted and delivered to a sample of participants with vision impairment to understand their sound localization difficulties compared to individuals with normal vision^25^. However, these tools do not assess visual localization with residual vision to facilitate direct comparisons between the two senses.

To address this gap, we developed the Dual Sensory Spatial Localization Questionnaire (DS-SLQ). As discussed above, we considered four questions to be critical for guiding individualized rehabilitation for DSI: (1) how good they judge their perceived vision localization ability to be, (2) how good they judge their perceived hearing localization ability to be, (3) which sense they judge to be their better sense for spatial localization, and (4) which sense they primarily rely on for spatial localization. The DS-SLQ uses a single structure to assess modality specific spatial localization abilities, which enables direct comparisons between vision and hearing. Each task in the questionnaire describes a real-life scenario that can be completed by either vision or hearing alone, and participants are prompted to report perceived difficulties in completing the task with each sense. Participants are also asked to provide judgments concerning the modality—vision or hearing—they would primarily rely on in each spatial localization scenario. Using modern psychometric analysis, we assess the validity of the DS-SLQ for providing direct comparisons between vision and hearing. We then use the DS-SLQ to investigate the differences in the perceived visual and auditory spatial localization abilities among four groups of participants with normal vision and normal hearing, vision impairment alone, hearing impairment alone, and DSI. Finally, we comment on the implications of our findings and the utility of the DS-SLQ in DSI rehabilitation.

## Method

### Spatial localization questionnaire

The DS-SLQ was developed by four co-authors (YX, JPN, WW, and GEL) who have expertise in low vision, DSI, O&M, and/or vision rehabilitation. DS-SLQ items were required to be: (1) related to spatial localization, which is defined as the ability to identify the direction and distance of people and objects around oneself; (2) common in everyday life; (3) tasks that necessitate the localization of objects or persons that can be localized by vision or hearing alone. In the first step, a large pool of potential tasks was aggregated and selected by YX from existing questionnaire instruments for vision (NEI VFQ^25,26^, Spatial Localization Questionnaire^13^, Visual Impairment Screening Questionnaire^27^), hearing (Speech Spatial Qualities^23^, American Academy of Otolaryngology-Head & Neck Surgery hearing test^28^, Hearing Handicap Inventory for the Elderly^29^), Activity of Daily Living, and Instrumental Activity of Daily Living^30^. In the second step, YX and GEL added tasks derived from their research observation and lived experience with low vision. In the third step, JPN and WW edited and included more tasks based on their clinical experience working with patients with low vision in O&M and vision rehabilitation services. Disagreements were actively discussed until consensus was reached.

After the initial development steps, the first draft of the DS-SLQ consisted of 50 tasks, which were reviewed by a sample of sixteen pilot participants with various combinations of vision and hearing impairment as well as by two O&M specialists who were not involved in the initial development of the DS-SLQ. Fifteen tasks were deleted due to being too complex to conceptualize (4 tasks), unusual (3 tasks), irrelevant (4 tasks, i.e., a direction/distance judgement is not required to complete the task), and repetitive (4 tasks). Slight rewording was applied to three items for clarification purposes. The revised DS-SLQ included 35 tasks, with 9 tasks related to safety, 8 to social, 8 to residential, and 10 to leisure tasks. For example, one of the safety related tasks is “You are walking along a city sidewalk. A police car is approaching with a siren sound and flashing light. You need to determine which direction the police car is from.” A full list of tasks is provided in Table 1.

**Table 1:** Task description and item measures.

Task	Category	Item measure (standard error), logits	
		Vision	Hearing
1. You are sitting in your home. A smoke detector goes off which flashes and makes an alarming sound. You want to determine where the fire alarm is	Safety	− 0.76 (0.16)	− 1.33 (0.19)
2. You are walking along a city sidewalk. A police car is approaching with a siren sound and flashing light. You need to determine which direction the police car is from	Safety	− 0.61 (0.18)	− 1.06 (0.29)
3. You are walking on a city sidewalk. There is a car passing by you on your parallel street to the side of you. Can you tell whether the car is accelerating or decelerating?	Safety	0.23 (0.14)	0.19 (0.15)
4. You are walking along a busy city sidewalk. Someone calls from across the street and waves to get your attention. You want to locate the person to respond	Safety	0.79 (0.13)	0.29 (0.14)
5. You are preparing to cross a street at an uncontrolled intersection. A car is approaching along the street from a distance. You need to decide if it is safe to cross before the car reaches you	Safety	− 0.15 (0.16)	1.26 (0.14)
6. You are walking in an outdoor parking lot where there are many parked cars. A car is pulling out of the parking lot and honks at you. You need to determine where the car is	Safety	0.27 (0.15)	− 0.53 (0.22)
7. During COVID, it is suggested that we keep at least 6 feet away from other people. You need to physically maintain this distance from others	Safety	− 1.06 (0.19)	1.31 (0.13)
8. You are approaching a moving walkway in the airport. The walkway has an alerting sound at its start point. You want to determine how far you are from the start point of the walkway	Safety	− 0.43 (0.16)	0.76 (0.13)
9. You are waiting for a bus at a bus stop. The bus stops and opens the door. You want to determine how far you are from the opening door	Safety	− 2.20 (0.33)	1.04 (0.13)
10. You are seated around a large round table with many people that you are meeting the first time. It is a quiet place. Someone begins to speak. Where is that person?	Social	0.10 (0.15)	− 0.27 (0.16)
11. You are sitting in the middle of two people. One of them is talking on their phone. Who is talking?	Social	− 0.61 (0.17)	− 1.28 (0.21)
12. You are sitting with two friends at a table in a busy restaurant. Your friends are in a discussion and are talking back and forth. You want to keep track of who is talking	Social	− 0.62 (0.19)	− 0.03 (0.15)
13. You are in the middle of a movie in a theater. Two people sitting somewhere are whispering to each other. You want to locate where they are	Social	2.18 (0.14)	0.82 (0.14)
14. You are in a party with many people. Your friend waves at you and calls your name. Where is your friend?	Social	0.64 (0.14)	0.66 (0.15)
15. You are in a big party. Someone raised their glass to toast to other people. You want to turn you head to face their direction	Social	0.01 (0.16)	0.67 (0.14)
16. You are walking in a hallway searching for the entrance of a conference room. Inside the conference room, there are people talking. How difficult is it to locate the room's entrance?	Social	− 0.38 (0.19)	0.23 (0.15)
17. You are sitting in a full conference room listening to a lecture. Somebody stands up and asks a question. You want to determine where that person is	Social	− 0.25 (0.16)	0.20 (0.15)
18. You are waiting for elevators in a building. There are three running elevators that make a beeping sound when they open up. You need to determine which elevator has opened	Residential	− 1.14 (0.21)	0.13 (0.17)
19. You are walking in a narrow hallway. Another person passes you from behind. You want to keep a distance from that person	Residential	− 0.48 (0.15)	0.70 (0.13)
20. You are walking in a hallway where there are doors on both sides. One of the doors suddenly closes and makes a sound. Is that door on your left or right side or the hallway?	Residential	0.34 (0.14)	− 0.13 (0.16)
21. You are in a hotel room. The phone rings and blinks. You want to pick up the phone	Residential	− 0.31 (0.16)	− 1.20 (0.23)
22. A child is holding a rubber ball and accidentally drop it on the hardwood floor. It bounces off in some direction. Can you find where the ball is?	Residential	0.08 (0.14)	1.18 (0.14)
23. You are standing in front of your window looking outside. Someone is shoveling snow; you want to find where that person is	Residential	− 0.59 (0.17)	0.99 (0.14)
24. You are taking a walk in your neighborhood. There is a dog running and barking. Where is the dog?	Residential	0.16 (0.14)	− 0.93 (0.20)
25. You are walking in a residential area. There are trees on both sides of the walkway. A bird is singing in the tree. Is the bird on your left or right side?	Residential	2.38 (0.15)	0.32 (0.14)
26. You are about to enter a shop in a shopping mall. A sales staff is standing at the front of the entrance and welcomes you. You want to avoid bumping into them	Leisure	− 2.08 (0.31)	0.35 (0.14)
27. You are in a clothing store in a shopping mall. There are people talking and moving at the checkout counter. You want to walk to the checkout counter	Leisure	− 0.92 (0.19)	0.97 (0.13)
28. You just entered a bathroom in a shopping mall. A cleaning staff is doing routine cleaning in one of the stalls. Can you tell which stall to avoid?	Leisure	− 0.85 (0.17)	0.50 (0.14)
29. You are entering a grocery store. The automatic door opens up as you approach and makes a sliding sound. You want to determine how far you are from that door	Leisure	− 1.44 (0.23)	0.35 (0.14)
30. You are shopping in a grocery store. Another customer is approaching with a shopping cart. You need to determine how far the customer is from you	Leisure	− 1.40 (0.21)	1.05 (0.13)
31. You are waiting to checkout in a grocery store. One of the staff waves at you and tells you that their counter is available for checkout. You want to determine where that person is	Leisure	− 0.41 (0.15)	0.48 (0.14)
32. A bird is flying overhead and chirping. You want to point it out to a friend	Leisure	0.77 (0.13)	0.54 (0.14)
33. You are sitting in a park. There are a couple bees flying around. You want to move away from the bees	Leisure	0.34 (0.13)	0.25 (0.15)
34. You are watching two people playing tennis in a tennis field. You want to track who is hitting the ball	Leisure	0.45 (0.13)	1.06 (0.13)
35. You are watching fireworks on July 4th. You want to locate the fireworks as they go off	Leisure	− 1.48 (0.22)	− 0.08 (0.16)

The final 35-task DS-SLQ was implemented as an online survey tool on Qualtrics and delivered through Zoom videoconferencing or a phone call according to participants’ preference. During the survey, participants verbally responded to the questions and the researcher registered the answers on Qualtrics. For each of the 35 tasks participants were asked (1) “How difficult it is to complete this task solely by your current vision?”, (2) “How difficult it is to complete this task solely by your current hearing?”, and (3) “Would you primarily rely on your vision or hearing for this task?”. Participants were instructed to provide the answers based on their habitual vision with or without corrections and habitual hearing with or without hearing aids. Participants could respond “Not Applicable” if they were unfamiliar with the tasks or situation posed in the question. For the difficulty questions participants used a 1–5 Likert scale, with 1 representing “very easy” and 5 representing “very difficult”. Middle values were not explicitly coded.

### Participants

One hundred and four (seventy women) participants were recruited from the Retiree Volunteer Center at the University of Minnesota, the Minnesota Laboratory for Low Vision Research, and the Envision Low Vision Rehabilitation Center. The inclusion criteria for vision and hearing status were broad—from normal to severely impaired—because of our interest in validating the DS-SLQ in a wide-ranging population. Exclusion criteria included age < 18 years old, near-total blindness without form vision, and near-total deafness with no useful hearing. This study was approved by the University of Minnesota Institutional Review Board (STUDY00001360) and followed the Declaration of Helsinki. Verbal informed consent was acquired from each participant prior to their participation.

### Vision and hearing screening

All participants were asked the following questions: “Do you have a vision problem that cannot be corrected by glasses or contact lenses?” and “Do you have a hearing problem?”. They were separated into four groups based on their answers: a control group with normal vision and normal hearing (N = 31, mean age 48.6 years); vision impairment-only (VI) group (N = 30, mean age 55.7 years); hearing impairment-only (HI) group (N = 21, mean age 75.4 years); and DSI group (N = 22, mean age 70.9 years). The majority of participants had acquired vision and/or hearing impairment rather than congenital impairment (Table 2).

**Table 2:** Participant characteristics.

Group	Control	VI	HI	DSI
N	31	30	21	22
Age, mean (SD), years	48.6 (22.8)	55.8 (16.1)	75.4 (5.9)	71.0 (16.6)
Onset, congenital/acquired*	NA	9/21	2/19	2/20
Visual acuity, mean (SD), logMAR	− 0.04 (0.12)	0.78 (0.57)	0.02 (0.08)	0.70 (0.54)
Contrast sensitivity, mean (SD), logCS	2.00 (0.21)	1.33 (0.66)	1.86 (0.13)	1.10 (0.57)
Field impairment, central/peripheral/intact	NA	10/10/10	NA	8/7/7
Pure tone average,^†^ mean (SD), dB HL	4.85 (6.93)	7.14 (7.29)	33.52 (11.68)	28.37 (11.32)

*SD:* Standard deviation, *VI:* Vision impairment only, *HI:* Hearing impairment only, *DSI:* Dual sensory impairment, *NA:* not applicable because there was no impairment, *MAR:* minimum angle of resolution, *HL* Hearing level.

*Congenital refers to people who report having vision/hearing impairment since birth.

^†^Pure tone average across 0.5, 1, 2 and 4 kHz in the better ear without correction.

We endeavored to collect standard vision and hearing screening data to quantify the level of vision and hearing impairment of our participants. Among the 104 participants, visual acuity (VA) measured by the Lighthouse Distance Visual Acuity chart^31^ was available from 65 participants, contrast sensitivity measured by the Pelli-Robson Contrast Sensitivity Chart^32^ was available from 61 participants, and unaided pure-tone audiometry using an air conduction audiometer was available from 60 participants. Hearing threshold was calculated as the better ear pure-tone average threshold across 0.5, 1, 2 and 4 K Hz in dB hearing level (HL) according to the World Health Organization standards^33^. Vision and hearing screening data were not available from the remaining participants because a screening visit was not practical due to travel or geographical limitations. Visual field status was self-reported as central field impairment, peripheral field impairment, or no field impairment. Table 2 summarizes the distributions of vision and hearing screening data in each group.

### Data analysis

All analysis was performed in R^34^. Rating scale data were analyzed with the method of successive dichotomizations (MSD)^35^ using the R package “msd”. MSD is a polytomous Rasch model that estimates ordered rating category thresholds, which makes it appropriate for analyzing rating scale data. The raw rating scale data were reversed in polarity prior to applying MSD (1 to 5 was flipped to 5 to 1) so that higher numbers correspond to better spatial localization abilities. MSD returns two primary outputs: item measures that represent the relative difficulty of each item (higher item measures representing more difficult items), and person measures that represent the relative spatial localization ability of each participant (higher person measures representing more capable individuals). Person and item measures are estimated on the same measurement scale in units of logit (log odds), so that the difference between a person and item measure translates to the probability that the person rates that item with a given rating category. A person with a higher person measure has a higher probability of rating any item as being less difficult, while an item with a higher item measure has a higher probability of being rated as more difficult by any person. Zero logit is by convention set to the mean item measure.

#### Psychometric validation

We first conducted modality-specific analysis with MSD: an analysis of the 35 vision difficulty ratings, and a separate analysis of the 35 hearing difficulty ratings. Our goal was to confirm that the 35 tasks of the DS-SLQ, whether used for vision alone or hearing alone, satisfied the key psychometric property of unidimensionality, i.e., all items in the questionnaire measured the same construct. Our test for unidimensionality was the percentage of information-weighted mean square fit (infit) statistics between 0.5 and 1.5^36^. Modality-specific analysis also allowed us to examine the correlation between vision and hearing item measures.

To directly compare vision and hearing person measures, we applied MSD to both vision and hearing difficulty ratings (70 items). First, we looked at the infit distribution to confirm that combining vision and hearing ratings preserved unidimensionality—whether there was a common latent variable for spatial localization measured by both vision and hearing items. Second, we compared person and item measure distributions to assess item targeting—whether item measures covered the full range of spatial localization abilities in our sample, and their density at different points of the scale was sufficient to discriminate between spatial localization abilities for all persons. Ideally, the distribution of item measures mirrors that of the person measures so that the greatest discriminability is achieved where most person measures lie. Poor item targeting may lead to a ceiling or floor effect, making it difficult to discriminate among individuals with good spatial localization ability (ceiling effect) or poor spatial localization ability (floor effect).

#### Spatial localization difficulties and primary sense

Next, we investigated differences in spatial localization ability when using vision alone versus using hearing alone for different groups of participants: Control, VI, HI, and DSI. For this analysis, we first estimated item measures from the combined analysis with DS-SLQ (i.e., 70-item: 35 for vision and 35 for hearing). Then, anchoring (or fixing) these item measures, we estimated separate vision and hearing person measures. This approach ensured that estimated vision and hearing person measures were on the same measurement scale and directly comparable.

We performed linear regression to determine whether there was a significant effect of “group” (Control, VI, HI, DSI) on vision person measures and hearing person measures. Age was included as a covariate because the groups differed significantly in their age (F (3,99) = 13.64, p < 0.001, η^2^ = 0.29). Pairwise t-tests with Bonferroni correction were conducted to examine the differences between each pair of groups when a significant main effect of “group” was observed (“emmeans” package^37^). Effect sizes are reported using η^2^ and Cohen’s d throughout the results.

We then asked which of the standard clinical vision and hearing assessments could explain observed group differences. To this end, we first performed univariate linear regression on the vision and hearing person measures to examine which of visual acuity, contrast sensitivity, visual field status (central/peripheral/no field loss), onset of vision impairment, binaural threshold, and onset of hearing impairment were significantly associated with the person measures. Significant factors were then incorporated in a multivariate linear regression to examine their joint contributions.

Lastly, we investigated whether participants’ self-report of primary sense was correlated with higher person measures for their better sense. To this end, we defined each participant’s “vision reliance” as the percentage of tasks reported to be primarily relying on vision, and their “vision advantage” over hearing as the difference between the vision and hearing person measures. We then examined the correlation between vision reliance and vision advantage.

## Results

### Psychometric validation

**Figure 1:**
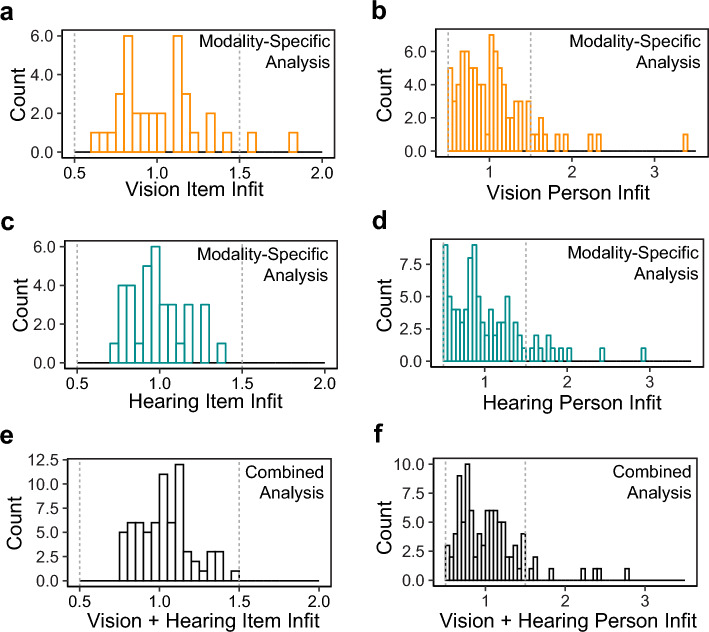
Histograms showing item and person infit of the modality-specific vision analysis (**a**, **b**), modality-specific hearing analysis (**c**, **d**), and the combined analysis (**e**, **f**). The vertical dashed lines mark the desired range of 0.5–1.5 for confirming unidimensionality of the DS-SLQ.

Figure 1a–d show the distributions of the person and item infits in the modality-specific analysis of vision and hearing ratings: 94.5% and 100% of the item infit, and 75.0% and 76.0% of the person infit, respectively, were within the desired range of 0.5–1.5. These observations provide evidence of unidimensionality for the DS-SLQ when only vision or only hearing is used for spatial localization. Figure 1e,f show the distributions of the person and item infits in the combined analysis: 100% of the item infit and 84.6% of the person infit were within the desired range of 0.5–1.5. This result indicates that there is a common latent trait for spatial localization that is shared by the two modalities.

**Figure 2:**
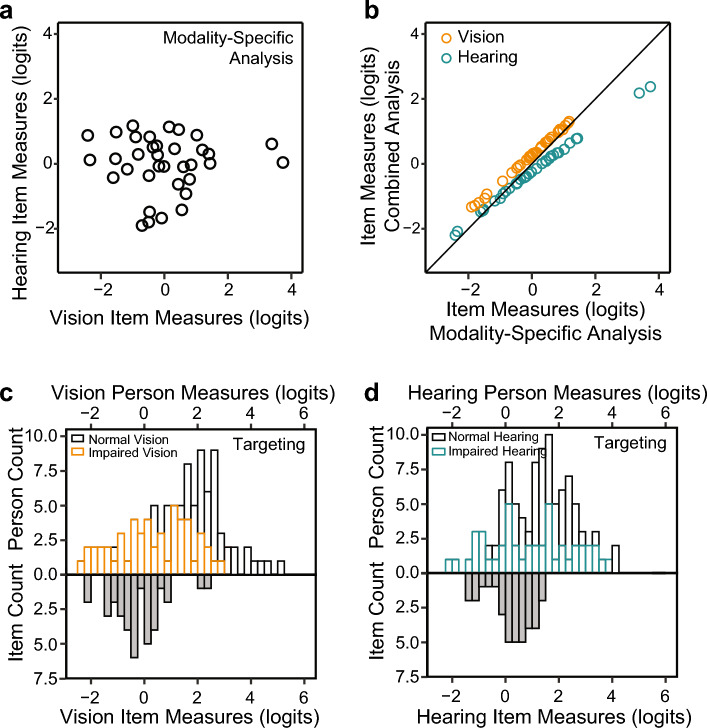
Item measures and targeting. (**a**) Scatter plot comparing modality-specific hearing item measures (y-axis) to modality-specific vision item measures (x-axis). (**b**) Scatter plot comparing vision (orange circles) and hearing (cyan circles) item measures from the combined analysis to their modality-specific counterparts. (**c**) Histogram of combined analysis vision person measures (top part) compared to histograms of item measures for normal (black bars) and impaired (orange bars) vision. (**d**) Histogram of combined analysis hearing person measures (top part) compared to histograms of item measures for normal (black bars) and impaired (blue bars) hearing.

Figure 2a shows that there was no significant correlation between modality-specific vision and hearing item measures (r = − 0.004, p = 0.98). This analysis shows that the difficulty of performing a spatial localization task in the DS-SLQ using vision alone is uncorrelated with the difficulty of performing that same task using hearing alone. Figure 2b shows that the relationship between item measures estimated from the modality-specific and combined analysis is approximately linear with the slope very close to 1 (the identity line). Thus, the combined analysis largely preserves the ordering of the item measures estimated from the modality-specific analysis.

Next, we examined item targeting of the DS-SLQ, i.e., how well the distribution of item measures mirrors the distribution of person measures in our sample. In Fig. 2c,d, we coded the person abilities using different colors, with orange for vision impairment, cyan for hearing impairment, and black for normal vision/hearing. For the participant pool in our current study, vision and hearing item measures are distributed toward the lower end of person measures, i.e., better targeted towards individuals with greater levels of impairment. Thus, while the infit statistic shows that the DS-SLQ measures a single underlying construct related to spatial localization, the DS-SLQ is primarily useful for individuals with impaired spatial localization. Using the DS-SLQ on individuals with normal vision and hearing leads to a ceiling effect. The vision and hearing person measures ranged from − 2.30 to 5.12 logits and from − 2.16 to 4.18 logits, respectively. The vision and hearing item measures ranged from − 2.20 to 2.38 logits and from − 1.33 to 1.31 logits, respectively.

Table 1 provides item measures and standard errors for each item. For vision, it is the easiest to judge the distance to a bus door (− 2.16 logits), and most difficult to locate a bird in a tree (2.38 logits). For hearing, it is the easiest to locate a smoke detector at home (− 1.33 logits), and most difficult to maintain social distancing from people during COVID-19 (1.31 logits).

For both vision and hearing, linear regression models on the item measures showed no significant differences (all p-values > 0.05) between item measures across the four categories of tasks (i.e., safety, social, residential, leisure).

### Vision and hearing person measures

**Figure 3:**
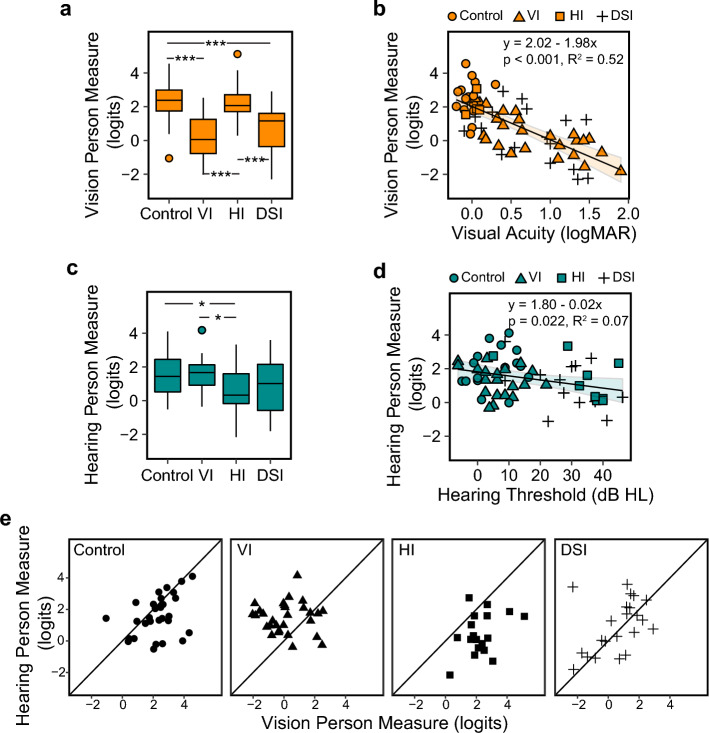
Vision and hearing person measures. (**a**) A boxplot showing the distributions of the vision person measures (in logit) across Control, VI, HI and DSI groups. (**b**) A scatter plot showing the correlation between the vision person measures and visual acuity in logMAR. The equations of the regression lines are provided in the plots. Individual data points from the four groups were illustrated using different symbols. (**c**) A boxplot showing the distributions of the hearing person measures (in logits) across the four groups. (**d**) A Scatter plot showing the correlation between the hearing person measures and better ear pure-tone hearing thresholds in dB Hearing Level. Regression line and the equation are also provided in the plot. Individual data points from the four groups were illustrated using different symbols. (**e**) Scatter plots showing the comparisons between the hearing and vision person measures in each of the four groups. *p < 0.05; ***p < 0.001.

Figure 3a shows the distributions of vision person measures for the four groups. There was a significant group difference (F(3,95) = 17.80, p < 0.001, η^2^ = 0.36), with the Control and HI groups showing significantly higher vision person measures (higher visual spatial ability) than the VI and DSI groups (p-values < 0.001, d ranged from 1.23 to 1.55). There was no significant difference (p-values > 0.05) between Control and HI, or between HI and DSI.

Visual acuity, contrast sensitivity, field impairment status, onset of vision impairment, but not hearing threshold or onset of hearing impairment, were significantly correlated with vision person measures. However, in the multivariate linear regression model, only visual acuity was a significant predictor (F(1, 61) = 67.4, p < 0.001, η^2^ = 0.52), explaining 51.7% of the variance in the person measures. As shown in Fig. 3b, each 0.1 logMAR (one line on the acuity chart) of worsening in visual acuity predicts 0.2 logits of decrease in the visual spatial ability.

Figure 3c shows the distributions of hearing person measures in each group. The four groups differed significantly in their hearing person measures (F(3,95) = 4.30, p = 0.007, partial η^2^ = 0.12), due to significantly lower hearing person measures in the HI group compared to the Control and VI groups (by 1.37 and 1.17 logits, d = 0.90 and 0.82, ps < 0.05). The DSI group also had lower person measures than Control and VI (by 1.01 and 0.81 logits, d = 0.69 and 0.58, respectively), although the differences were not statistically significant (p = 0.09 and 0.24, respectively). There was no significant difference between Control and HI, or between VI and DSI (p-values > 0.05).

Hearing threshold, but not onset of hearing impairment or any of the vision screening parameters, was a significant predictor for the hearing person measure (F (1,56) = 5.52, p = 0.022, η^2^ = 0.09), although it only explained 7.34% of the variance in hearing person measures. As shown in Fig. 3d, each 10 dB increase in hearing threshold was associated with 0.24 logits decrease in the hearing spatial ability.

### Self-reported primary sense

When comparing vision and hearing person measures in each of the four groups, most participants in the Control group showed vision as their “better sense” with a higher vision person measure than hearing (mean difference = 0.67 logits, p = 0.016, d = 0.45, Fig. 3e). This result is consistent with the classic vision advantage in spatial tasks^8,15,19–21^. This vision advantage changes with both vision and hearing impairment. For the VI group, the vision advantage was reduced, with the majority of the participants showing a higher hearing ability than visual ability (mean difference = − 1.35 logits, p < 0.001, d = 1.35). For the HI group, the vision advantage was greater than in the Control group, with all but one participant showing higher vision ability than hearing ability (mean difference = 1.83 logits, p < 0.001, d = 0.79). For the DSI group, the results were less clear. As shown in Fig. 3e, many of the DSI participants had comparable vision and hearing person abilities (mean difference = − 0.35 logits, p = 0.33, d = 0.21).

**Figure 4:**
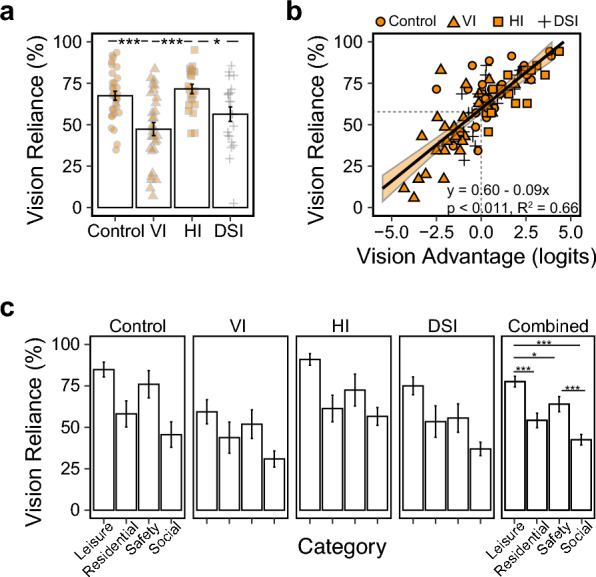
Vision reliance patterns. (**a**) A bar plot showing the average percentage of tasks reported as “primarily relying on vision”, for each of the four groups. Individual datapoints are also illustrated as an overlay on the bars. (**b**) A scatter plot showing the correlation between vision reliance and vision advantage. For each participant, vision advantage was calculated as the difference between the vision person measure and hearing person measure. (**c**) Bar plots showing vision reliance in each of the four categories. The right-most panel shows the combined results across all participants in each category. *p < 0.05; ***p < 0.001.

If participants’ self-report of “primary sense” is consistent with their better sense indicated by the person measures, we would expect the vision reliance across tasks to be highest in the HI group, followed by the Control, DSI, and lowest in the VI group. This prediction was supported, as shown in Fig. 4a. The HI group had a vision reliance (mean ± SD) of 71.6% ± 4.0%, followed by the Control (67.5% ± 3.3%), DSI (56.4% ± 3.9%), and lastly, the VI (47.3% ± 3.3%) group. There were significant differences (F(3,100) = 9.79, p < 0.001, η^2^ = 0.23) between the HI and both DSI and VI groups (ps < 0.05, d = 0.86 and 1.33), and between the Control and VI groups (p < 0.001, d = 1.11).

Figure 4b further visualizes the correlation between vision reliance across tasks and vision advantage in person measures. As vision advantage increases, vision reliance increased in a linear manner (F (1, 96) = 193.16, P < 0.001, η^2^ = 0.67). Each 1 logit of increase in vision advantage was associated with an 8.9% increase in vision reliance (i.e., three more tasks reported as primarily relying on vision). When the vision advantage was 0 (comparable vision and hearing person measures), vision reliance was 59.6%, indicating a bias towards relying on vision.

Lastly, we compared vision reliance across the four task categories (i.e., safety, social, residential, leisure). We found a significant main effect of category (F(3,124) = 17.26, p < 0.001, partial η^2^ = 0.29) that was consistent across all groups despite the group differences in the overall vision reliance: the leisure related tasks had the highest vision reliance, followed by safety and residential tasks, and lowest in the social tasks (Fig. 4c).

## Discussion

We developed a Dual Sensory Spatial Localization Questionnaire (DS-SLQ) to investigate the potential effects of combined vision and hearing impairments on the perceived difficulties of real-life spatial localization tasks. Through modern psychometric analysis^35^, we showed that the DS-SLQ assesses a unidimensional latent variable underlying both vision and hearing spatial localization abilities. We also showed that the DS-SLQ is well targeted to individuals with low spatial localization abilities. To our knowledge, this is the first questionnaire that provides simultaneous assessment and direct comparison between vision and hearing spatial abilities in people with DSI.

Using the DS-SLQ, we confirmed that vision impairment and hearing impairment were each associated with reduced visual and auditory spatial localization abilities. For vision person measures estimated from the DS-SLQ, acuity explained the most variance across individuals. However, acuity has been reported in previous studies to be a weak predictor for performance-based measures of spatial localization tasks, such as navigating mobility courses and simple direction judgement tasks^15,38^. This discrepancy is possibly due to acuity playing a more significant role in subjective evaluation than in performance measures^39^. It should also be noted that our questionnaire included a variety of everyday visual targets with different angular sizes from typical viewpoints, which may be more sensitive to acuity impairment than simple visual objects in laboratory tasks. Hearing impairment, measured as thresholds, only weakly affected the hearing person ability measured by DS-SLQ, which was consistent with the literature^40,41^. Note that the hearing thresholds were measured without hearing aids following standard practice, while the hearing ability as assessed by DS-SLQ asked participants to consider their habitual use of hearing aids, if any. This may have contributed to a weaker correlation between the thresholds and reported hearing abilities.

The design of the DS-SLQ is motivated by the classic behavioral paradigm for investigating sensory integration in spatial localization, in which participants were asked to localize visual, auditory, and bimodal conditions^20,21,42–44^. The bimodal condition was used to compute the relative contributions of vision and hearing to the localization of bimodal tasks. A dominant model for sensory integration is that the sense with higher reliability would have higher contributions to the bimodal condition^21,43,44^. The DS-SLQ focuses on the self-reported difficulties in various everyday localization tasks, which is distinct from behavioral studies in simple laboratory environment. However, we were able to translate the merit of the behavioral design to obtain modality-specific vision and hearing difficulty scores, as well as sensory reliance patterns. We found that a person’s vision reliance pattern, quantified as the percentage of tasks reported as being vision dominant, is largely explainable by the difference between visual and auditory person abilities. As the advantage of vision over hearing increase, vision reliance increases. Humans appear to have a general cognitive and sensory ability to make sensory dominance decisions based on their own abilities. Moreover, the DSI group appeared to have the most symmetric vision and hearing abilities (Fig. 3) and may benefit the most from integrating vision and hearing as predicted by optimal sensory integration models.

**Figure 5:**
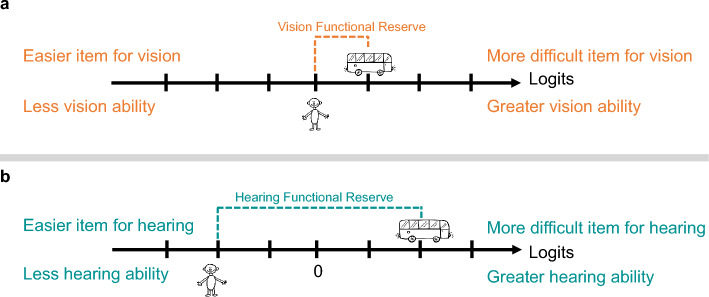
A hypothetical case showing the rehabilitation utility of DS-SLQ. A patient with DSI is seeking Orientation and Mobility training for intendent travel, in which one of their goals is to successfully locate the opening door of a bus. Through DS-SLQ the therapist obtained the person’s vision and hearing person measures. (**a**) By comparing the vision person measure to the vision item measure, the therapist obtains the vision functional reserve. (**b**) By comparing the hearing person measure to the hearing item measure, the therapist obtains the hearing functional reserve. By comparing the vision and hearing functional reserves, the therapist may communicate with the patient about prioritizing vision training for this specific task.

In the context of rehabilitation, we consider the DS-SLQ an informative tool for the initial and final assessments for people with sensory impairment, especially for those with DSI. In Orientation and Mobility training, vision and hearing person measures estimated using the DS-SLQ can help a therapist understand an individual’s relative vision and hearing spatial abilities on the same measurement scale. If the relative ability is inconsistent with the individual’s sensory reliance preference, this information can be provided to the individual to help them adjust their spatial localization strategy. By comparing person measures to item measures, a therapist can identify tasks that are beyond the person’s vision and hearing abilities, within a person’s vision ability but not within that person’s hearing ability (or vice versa), or within both vision and hearing abilities. For a given task, the difference between the person measure and item measure is called “functional reserve”^45^. Visual and auditory functional reserves can help a rehabilitation professional together with their client develop an individualized training plan. Figure 5 illustrates a hypothetical scenario where a patient with DSI wishes to locate an opening door on a bus more effectively. Using the DS-SQL, the therapist finds that the functional reserve for vision in this task is quantitatively smaller (less negative) than for hearing. The therapist and the client may therefore decide to focus on vision training for this task.

The utility of the DS-SQL extends to hearing rehabilitation. In communications with our participants with DSI, a frequent complaint was the lack of sound directionality of hearing aids. As hearing aid technology advances to better accommodate directionality, the DS-SQL can help audiologists identify which patients may have difficulties using their vision to compensate for sound localization. For these patients, the audiologists could recommend a specific model or adjust the settings of their hearing aids to better convey sound directionality.

There are several limitations to the current version of the DS-SLQ as well as the current study. First, the current DS-SLQ is better targeted towards individuals with severe impairment and low spatial localization ability. To make the DS-SLQ more sensitive to mild impairment, more difficult tasks could be added, such as visual localization at night or under dim lighting. The current DS-SLQ was also not designed or validated using participant ratings of “overall difficulty” of each task, i.e., using both vision and hearing as well as any other senses. A limitation of the current study is that participants answered based on their habitual vision/hearing with or without vision or hearing aids (if any) to reflect their everyday experience. It would be useful to estimate the effect of vision and hearing aids as well as the added benefit of other mobility aids such as white canes and guide dogs. Another issue with the current DS-SLQ is that it takes 30–40 min to administer, which may be prohibitive in many clinical settings. A future study will investigate shortening the DS-SLQ by reducing the total number of items while preserving key psychometric properties such as targeting. Lastly, because the DS-SLQ was delivered over zoom to access a broad participant pool, it was challenging to obtain clinical vision and auditory data from many participants. In the future, it would be helpful to include validated remote vision and hearing screening tests with the DS-SLQ to better quantify the association between standard screening parameters and spatial localization ability.

Another important future direction is to compare performance-based measures with self-report in the DS-SLQ. The list of real-life tasks in the DS-SLQ and their associated vision and hearing task measures offer us a map for selecting representative performance tasks. For example, performance-based spatial localization tasks can be selected to include tasks that are more visually difficult, more auditorily difficult, both difficult, and both easy. Such tasks will help us understand the visual and auditory performance required to complete each task, the interactions between vision and hearing when relative abilities of the two senses change, and the benefit of sensory integration for different individuals under different real-life tasks. We are pursuing these future directions in the next stage of this research.

## Data Availability

We will make the de-identified raw data available upon request. Please contact Yingzi Xiong at yxiong36@jh.edu.
